# Performance status score: do patients and their oncologists agree?

**DOI:** 10.1038/sj.bjc.6601231

**Published:** 2003-09-09

**Authors:** S P Blagden, S C Charman, L D Sharples, L R A Magee, D Gilligan

**Affiliations:** 1 Department of Oncology, Box 193, Addenbrooke's Hospital, Hills Road, Cambridge CB2 2QQ, UK; 2 MRC Biostatistics Unit, Institute of Public Health, University Forvie Site, Robinson Way, Cambridge CB2 2SR, UK; 3 Thoracic Oncology Unit, Papworth Hospital, Cambridge CB3 8RE, UK

**Keywords:** ECOG, performance status, lung cancer, oncologist, prognosis

## Abstract

Oncologists traditionally assess their patients' ECOG performance status (PS), and few studies have evaluated the accuracy of these assessments. In this study, 101 patients attending a rapid access clinic at Papworth Hospital with a diagnosis of lung cancer were asked to assess their own ECOG PS score on a scale between 0 and 4. Patients' scores were compared to the PS assessment of them made by their oncologists. Of 98 patients with primary non-small-cell lung cancer (NSCLC) and small-cell lung cancer (SCLC), weighted *κ* statistics showed PS score agreement between patient and oncologist of 0.45. Both patient- and oncologist-assessed scores reflected survival duration (in NSCLC and SCLC) as well as disease stage (in NSCLC), with oncologist-assessed scores being only marginally more predictive of survival. There was no sex difference in patient assessment of PS scores, but oncologists scored female patients more pessimistically than males. This study showed that, with few exceptions, patients and oncologists assessed PS scores similarly. Although oncologists should continue to score PS objectively, it may benefit their clinical practice to involve their patients in these assessments.

## 

### Performance status

David A Karnofsky and colleagues described the first performance status (PS) score in 1948 (Karnofsky *et al*, 1948). It was introduced for assessing patients receiving nitrogen mustard chemotherapy for primary lung carcinoma. Each patient was given a score on a linear scale between 0 (dead) and 100 (normally active), summarising their ability to perform daily activities, and the level of assistance they required in order to do so. This scoring system was subsequently used throughout oncology practice as a numerical guide to patients' general health. In 1960, the Eastern Co-operative Oncology Group (ECOG) introduced a simpler ‘ECOG performance status’ scale, similar to the Karnofsky PS (KPS) scale, with only five points. This is now termed the ECOG/WHO score (Oken *et al*, 1982) having been expanded to comprise of six points with the addition of PS 5 (Figure 1

**Figure 1:**
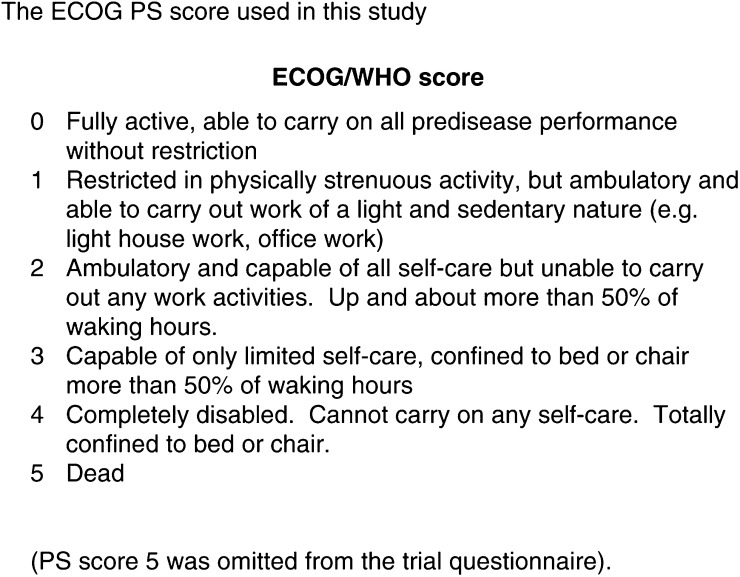
The ECOG PS score used in this study.

).

In a Medline Search using the terms ‘clinical trial and Karnofsky/WHO or ECOG performance status’, 233 and 84 authors used the ECOG and Karnofsky scores, respectively. Generally, the two scores have been proven to be interchangeable (Taylor *et al*, 1999), although the ECOG is often preferred for its simplicity.

### Interobserver agreement

A score is reliable if there is good concordance between observers, and low rates of inter- and intraobserver variability. Studies of this have been conflicting. When health professionals were asked to assess the KPS of 75 cancer patients, Yates *et al* (1980) found moderate agreement (correlation coefficient 0.69) between nurses and social workers. Oncologists and psychiatrists or psychologists had greater agreement with a correlation coefficient of 0.79. Roila *et al* (1991) showed very high interobserver correlation between two oncologists in assessing both the Karnofsky (coefficient 0.92) and ECOG (coefficient 0.91) scores in 209 cancer patients, but Sorenson *et al* (1993) showed only moderate overall nonchance agreement among three oncologists who assessed the ECOG status of 100 consecutive cancer patients seen in an outpatient clinic. They had higher concordance in patients with good PS (ECOG PS 0-2) compared to those with poor PS (ECOG PS 3-4).

### Correlation of performance status with other variables

Performance status scores are widely used in oncological practice because they correlate with patient survival duration (Albain *et al*, 1991) and response to treatment (Sengelov *et al*, 2000), as well as their quality of life (Finkelstein *et al*, 1988) and comorbidity (Firat *et al*, 2002). This scoring system is therefore used to decide which patients are physically suitable for treatment and/or entry into clinical trials. Many lung cancer chemotherapy trials have a cutoff of PS>1 because patients with PS=2 have been shown to have particularly poor outcome in clinical trials after treatment (Sweeney *et al*, 2001; Bunn, 2002).

A number of studies have measured the accuracy and reliability of both Karnofsky and ECOG scores. To assess accuracy, Mor *et al* (1984) demonstrated that the KPS of terminal cancer patients correlated well with patient longevity. Buccheri *et al* (1996) compared the KPS and ECOG PS in 536 patients with terminal cancer and found that the ECOG test was better than the Karnofsky score at predicting patient prognosis. Other studies have combined performance status with other variables (e.g. biological, proliferative and serum markers) to give a more specific method of scoring prognosis. There are now over 150 of such markers that can be used to predict prognosis for patients with lung cancer (Brundage *et al*, 2002), but PS is still the main (or only) prognostic score commonly used.

To investigate whether PS correlated with survival, the Edinburgh Lung Group (Capewell and Sudlow, 1990), conducted a study in which the Karnofsky score of 651 newly registered patients with lung cancer was assessed by physicians and then correlated to the patients' survival. In patients not treated surgically, those with a KPS>90 survived for a median 9.3 months, 6.2 months for an index of 80, 4.5 months for index 60–70 and 1.2 months for an index of 50 or less.

### Doctor–patient agreement

Performance scores are clearly very influential but are usually assessed by clinicians rather than by patients themselves. This is in contrast to quality of life scores, which are now subjectively assessed by patients themselves, since it was shown that they were more reliable & consistant at scoring their own quality of life than their doctors (Slevin *et al*, 1988). Perhaps patients should routinely be assessing not only their quality of life, but also their PS. This would at least overcome the problem of interobserver variability.

A study to investigate this was performed by Ando *et al* (2001) in Japan. In this study, 206 consecutive in-patients with stage III or IV non-small-cell lung cancer (NSCLC) were asked to assess their own PS and this was compared to an objective assessment by their oncologists. Surprisingly, there was only moderate agreement between the two groups, with patients being more pessimistic than their physicians. The agreement was particularly poor between female patients and their oncologists at 37.2% compared to 54% between male patients and their oncologists. Overall, they found that the oncologist-nominated PS correlated most closely to observed survival data. They concluded that oncologists were better at evaluating PS than the patients themselves.

The purpose of our study was to compare patient-assessed PS with that recorded by the treating oncologist at the first clinic appointment, before the patient was informed of their diagnosis. We also aimed to evaluate the relationship between PS scores and patient's sex, their tumour stage and subsequent survival duration. To measure ‘accuracy’, we wished to assess which PS (oncologist- or patient-assessed) was more closely associated with survival.

## MATERIALS AND METHODS

This was a prospective, nonrandomised, double-blinded, longitudinal study of the ECOG PS of patient seen consecutively, attending a single institution (Papworth Hospital) with a histological diagnosis of SCLC or NSCLC.

Between March 2000 and October 2001, 101 patients who attended the Papworth two-stop oncology clinic with a suspected diagnosis of lung cancer were prospectively entered into the study. Before being seen by an oncologist, and before the diagnosis of lung cancer was discussed, patients waiting in clinic were given an information sheet about the study and asked if they would like to take part. Those who agreed signed a consent form and were then required to assess their own ECOG PS in a questionnaire by placing a tick beside the statement that most accurately reflected their functional ability over the preceding 2 weeks (Figure 1). Following their clinic consultation, the oncologist recorded the patient's ECOG score using a similar questionnaire. Oncologists participating in the study were requested to assess PS using their usual clinical practice.

Once the patient and oncologist had completed the PS forms, the research nurse took note of the patient's age, sex, type and stage of disease and the treatment plan. Both patient and oncologist were blinded to the other's PS assessment, and patients were asked not to inform the oncologist of their selected score during the consultation. The patient was then treated in the usual manner, that is, with radiotherapy, chemotherapy, surgery or supportive care according to the stage and type of their disease and their clinician-assessed PS.

The research nurse monitored the outcome of patients that had entered this study. Of those who died within 2 years of study commencement, their date of death was recorded from the Hospital Patient Administration System. This study met with LREC approval.

## STATISTICS

Agreement between patient and oncologist assigned-PS scores was assessed using weighted *κ* statistics and reported with 95% confidence intervals (95% CI). Pearson's *χ*^2^ test was used to test whether PS scores were associated with stage (NSCLC) or with sex. Survival was calculated in months and days from date of trial entry and initial PS assessment (usually the same as date of pathological diagnosis) to the date of death, or 20 June 2002 if alive. Actuarial survival was estimated using Kaplan–Meier methods and reported as median (interquartile range (IQR)) or cumulative survival (95% CI). Between-group comparisons were made using the log-rank test. Univariate Cox's regression was used to evaluate which score (patient- or oncologist-assessed) was a better fit to the observed survival data. The value of −2 × log likelihood (-2LL) was reported for each model: lowers values giving better fit. Multivariate Cox regression was also used to evaluate the association of PS score (patient- or oncologist-assessed) with survival (for NSCLC and SCLC), adjusting for sex and stage of disease (in NSCLC only). All three variables were entered into the model as categorical variables. The significance of PS score was assessed by the likelihood ratio test on removal from the saturated model. The −2LL was also reported for these adjusted models.

## RESULTS

In total, 10 oncologists took part in this study, consisting of two consultants and eight specialist registrars. The majority of patients were male (71%); the median age was 70 years (range 34–88 years). All patients had a diagnosis of a malignancy, 81 with NSCLC, 17 with SCLC and three who were subsequently found to have secondary lung metastases from a non-lung primary. These three patients were excluded from the subsequent survival analysis, so that of the remaining 98 patients with a primary diagnosis of lung cancer, the median (IQR) survival time was 7.7 months (3.7,17) and at study completion, 20 patients (20%) were still alive.

### SCLC

Patients with SCLC were staged as limited or extensive disease (Table 1

**Table 1:** Disease type and stage of 98 patients with primary lung cancer in this study

**Diagnosis**	**Disease stage**							
Non-small-cell lung cancer 81	Ia 5 (6%)	Ib 13 (16%)	IIa 2 (3%)	IIb 2 (3%)	IIIa 14 (17%)	IIIb 25 (31%)	IV 19 (23%)	u/k 1 (1%)
Small-cell lung cancer 17	Limited stage 11 (65%)				Extensive stage 6 (35%)			

u/k=unknown.

). The majority (65%) of the 17 patients in this study with SCLC had limited disease on diagnosis. Overall, their median (IQR) survival from trial entry was 6.5 (2.2,12) months. As the number of patients with SCLC in this study was small, stage-by-stage analysis was not performed on them.

### NSCLC

**Figure 2:**
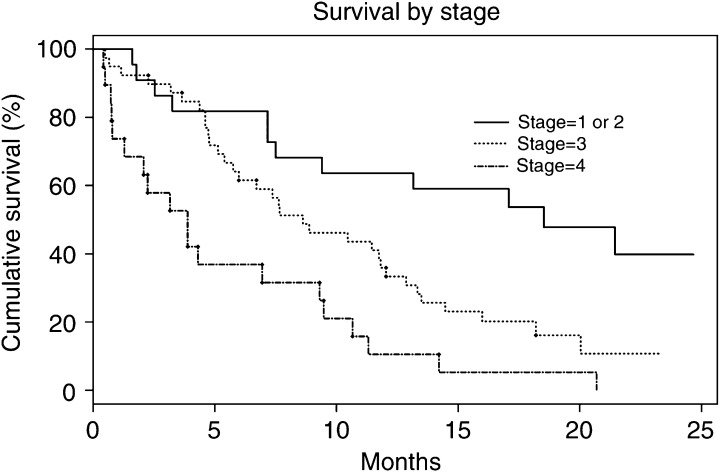
Kaplan–Meier graph showing cumulative survival of 81 study patients with a diagnosis of NSCLC according to the pathological stage of their disease at diagnosis. Survival taken from date of trial entry until death or completion of study (if alive).

Of 81 patients with NSCLC, 72% had stage III or IV disease on diagnosis (Table 1). The overall actuarial survival (95% CI) at 1 year from trial entry was 35% (25, 46). The 1-year survival rates by stage were 64% (43, 85), 33% (18, 48), 11% (0, 24) for stages I or II, III and IV, respectively (Figure 2

).

### Correlation of PS with survival

Performance status, whether assessed by oncologist or patient, was significantly associated with survival in the 98 patients with primary lung cancer (*P*⩽0.001). (Tables 2

**Table 2:** Survival by performance status (NSCLC and SCLC) assessed by (a) patient^a^ and (b) oncologist^b^

**Score**	**Median survival (months)**	**Cumulative survival (95% CI) at 1 year**
(a) *Patient*		
0	12.9	52% (31, 73)
1	11.3	40% (25, 55)
2	6.5	17% (2, 33)
3–4	2.2	14% (0, 32)

(b) *Doctor*		
0	18.9	67% (44, 88)
1	8.2	41% (26, 57)
2	6.5	17% (2, 35)
3–4	2.1	17% (0, 38)

**Figure 3:**
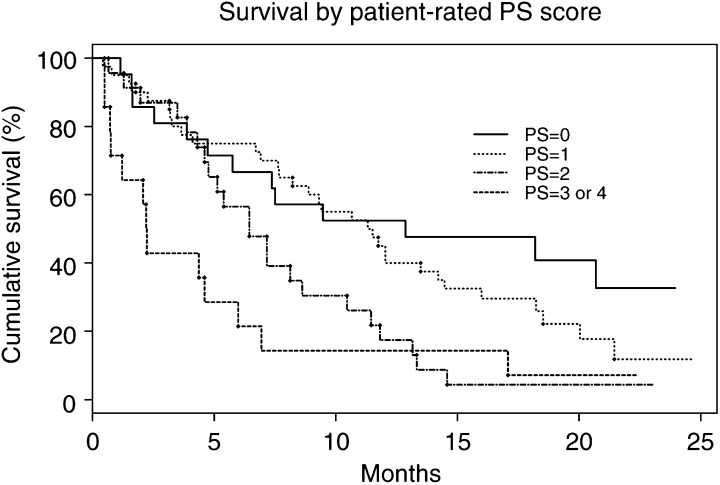
Kaplan–Meier graph showing cumulative survival in patients with NSCLC according to their patient-assessed PS scores (data: Table 2). Survival correlated with PS, *P*=0.001.

**Figure 4:**
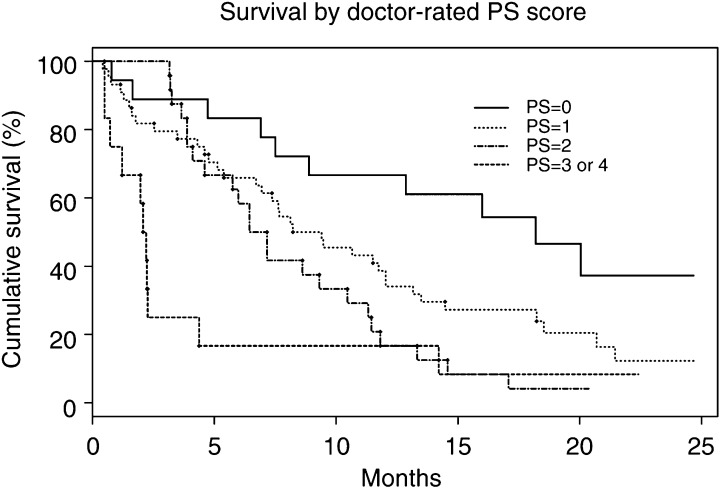
Kaplan–Meier graph showing cumulative survival in patients with NSCLC according to their oncologist-assessed performance status scores (data: Table 2). Survival correlated with PS, *P*<0.001.

In both cases, performance status correlated with survival (*P*⩽0.001). This is represented graphically in Figures 3 and 4. NSCLC=non-small-cell lung cancer; SCLC=small-cell lung cancer; CI=confidence interval.

a Log rank test *P*=0.001.

b Log rank test *P*<0.001.

and 3

**Table 3:** Differences in oncologist- and patient-assessed performance status (PS) scores in relation to disease stage, type and sex of patient

	**Doctor-assessed PS score compared to patient assessed PS score**		
**Group/subgroup (*n*)**	**Under-rated (D<P) *n* (%)**	**Agree (D=P) *n* (%)**	**Over-rated (D>P) *n* (%)**
All patients (101)	27 (27)	51 (50)	23 (23)
Females (29)	3 (10)	16 (55)	10 (35)
Males (72)	24 (33)	35 (49)	13 (18)

NSCLC			
Stage unknown (1)	1 (100)	–	–
Stage I (18)	5 (28)	9 (50)	4 (22)
Stage II (4)	1 (25)	3 (75)	–
Stage III (39)	11 (28)	19 (49)	9 (23)
Stage IV (19)	6 (32)	6 (32)	7 (36)

SCLC			
Limited (11)	1 (9)	8 (73)	2 (18)
Extensive (6)	1 (17)	4 (66)	1 (17)

Stage of NSCLC was not associated with PS score, whether assessed by patient or oncologist (*P*=0.18). Although there was no statistical difference in PS scores between males and females (*P*=0.37), oncologists generally gave lower PS scores to female patients (*P*=0.04, marginally significant). D=doctor-assessed PS score; P=patient-assessed PS score; NSCLC=non-small-cell lung cancer; SCLC=small-cell lung cancer.

, Figures 3

and 4

). Multivariate Cox's regression showed that PS score was significantly associated with survival in these patients, after adjustment for sex (NSCLC and SCLC) and stage (NSCLC) (patient score *P*=0.005; doctor score *P*=0.007).

### Doctor–patient agreement

The patient- and oncologist-assessed PS scores are given in Table 4

**Table 4:** Performance status (PS) scores (NSCLC and SCLC) as assessed by patient (vertical axis) and oncologist (horizontal axis)

	**ONCOLOGIST**					
	**Score**	**0**	**1**	**2**	**3**	**4**
**PATIENT**	**0**	*10*	10	2	0	0
	**1**	10	*22*	8	2	0
	**2**	0	11	*11*	1	0
	**3**	0	2	3	*8*	0
	**4**	0	0	0	1	*0*

Numbers underlined are cases when oncologist and patient agreed over PS score. Overall, weighted *κ* score=0.45 (0.33, 0.59) indicating moderate agreement between patient- and oncologist-assessed scores.

. Patient and oncologist agreed in 51 (50%) of the cases. The weighted *κ* (95% CI) was 0.45 (0.33, 0.59) indicating moderate agreement; they were generally similar in their PS assessment, but patients were marginally less optimistic than their oncologists. In cases where they disagreed, the PS scores were evenly spread with 46% of patients giving higher (optimistic) and 54% giving lower (pessimistic) PS scores than their oncologists. There were only six cases when PS scores differed by more than one point (see Table 5

**Table 5:** Characteristics of the six patients who differed in subjective and objective performance status scores by 2 or more points

**Patient**	**Sex**	**Disease stage (NSCLC)**	**Survival (days)**	**Patient assessed score**	**Doctor-assessed score**
1	Male	III	69	1	3
2	Male	III	175	0	2
3	Female	IV	118	0	2
4	Female	IV	432	1	3
5	Male	IV	23	3	1
6	Male	IV	211	3	1

).

In the 12 cases where the patient scored themselves as PS 2, 11 (92%) patients were scored more optimistically by their oncologist (i.e. given a score of 1 instead of 2). Although one patient did score himself as PS 4, we would not expect patients of low PS in this study as they were recruited as outpatients, and required mobility to attend.

### Sex difference and disease stage in PS assessment

There was no significant difference in the patient-assessed PS scores when comparing males with females (*P*=0.37), with weighted *κ* scores of 0.4 (0.24,0.55) and 0.57 (0.32, 0.81) for males and females, respectively. However, on average, oncologists gave lower scores to females more often than males (*P*=0.04; marginally significant).

Stage of disease (NSCLC only) was not associated with PS score, whether assessed by patient or oncologist (both *P*=0.18). The distribution of patient – oncologist scores is given by stage of disease in Table 4.

### ‘Accuracy’ of PS

To calculate which PS score (patient- or oncologist-assessed) was the better fit to the observed survival data, a Cox regression model was run with each score separately. For the univariate models the values of −2LL were 615.1 and 612.7 for patient and oncologist, respectively. Their corresponding values in the adjusted models were 446.9 and 447.6. Both sets of figures indicated that scores fit the observed data to a similar extent.

## DISCUSSION

### Correlation of PS with survival

Both patient- and oncologist-assessed scores correlated with survival duration (Figures 3 and 4) confirming the findings of other studies (Mor *et al*, 1984) that have validated PS as a reliable prognostic marker in patients with cancer. Even allowing for sex and stage of disease, patient- and oncologist-allocated PS scores were independently associated with survival.

### Doctor–patient agreement

We demonstrated moderate agreement between patients and oncologists in their assessments of patients' PS (weighted *κ* 0.45). There was an even spread of PS scores, with patients and oncologists rarely disagreeing by more than one PS point. This is in contrast to the study by Ando *et al* (2001), which, although demonstrating a higher incidence of agreements between oncologist and patient (*κ* score 0.53), had wider disparity of scores in those that did not agree. This occurred particularly in female patients, who scored themselves more pessimistically than their male counterparts. In our study, although there was no statistical difference in subjective PS scoring between male and female patients, our oncologists tended to score the female patients more pessimistically than the males. The contrast in results between the Ando study in Japan and ours could reflect different cultural perceptions of illness or variations in study design: the Ando study was carried out on sequential in-patients with NSCLC, whereas ours was performed in the two-stop outpatient clinic with patients who were awaiting confirmation of a primary lung cancer diagnosis.

The even spread of PS scores in our results was belied by the *κ* score that showed only moderate agreement between oncologist and patient. The greatest area of disagreement appeared to be over the assignation of PS score 2 with oncologists often choosing PS=0 or 1 instead.

### Disease stage and PS

Here, 10 oncology registrars or consultants were involved, which may have caused bias, as they were of different sex, age and clinical experience. However, we designed this trial to reflect current practice in the lung cancer clinic and to reduce potential assessment-bias from an individual doctor in PS allocation. Participating oncologists had the benefit of reading patients' notes and viewing staging test results before allocating a PS score and this could have influenced their assessments. In a trial of this design, it would be difficult to demonstrate whether or not this occurred. However, we showed no statistical correlation between oncologist-assigned PS and stage of disease (*P*=0.18), indicating that they were not using disease stage to guide their assessment. If this had been the case, patients with more extensive disease would have been assigned lower PS scores.

**Figure 5:**
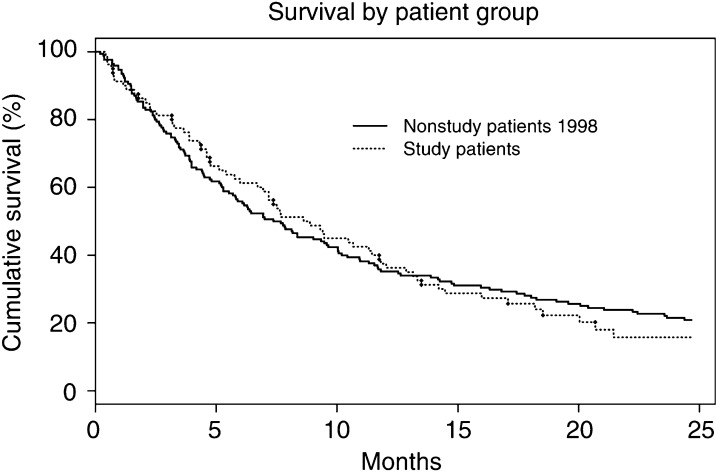
Overall survival of 170 (nonstudy) patients with NSCLC enrolled in the two-stop clinic in 1998 compared with survival of 81 (study) patients with NSCLC. There was no statistical difference in survival between them. *P*=0.83.

Also, we compared the survival of patients with NSCLC in this study to that of 170 similar patients seen in the two-stop clinic in 1998. This was to show that patients in our study were typical of those seen in the two-stop clinic, and were managed according to usual clinical practice. In the 1998 group, 1-year survival was 35% (28%, 42%). There was no significant difference (*P*=0.77) in overall survival, adjusted for stage, between the 1998 patients and those in our study (Figure 5

).

### ‘Accuracy’ of PS assessment

So which PS scores showed greatest prognostic accuracy, those decided by the patients or by their oncologists? Comparing the PS-related survival of patients in the oncologist-assessed, and then the patient-assessed groups to that recorded in other studies would address this. However, despite the fact that PS scores are known to correlate to survival, few studies have quantified the expected survival for each PS score in NSCLC. Survival differs for every type of cancer, and in the few studies where it is quantified; the PS scores have always been doctor-assessed (Buccheri *et al*, 1996). However, using the univariate Cox's regression model, we showed that, within the limits of this study, patient- and oncologist-assessed PS score reflected survival to a similar degree.

An important criticism of this type of study is that bias may be introduced if treatment is assigned to patients on the basis of the oncologist-assessed PS score. Thus, if an oncologist gave a fit patient a PS of 3, the patient would then receive supportive care rather than active treatment (with surgery, chemotherapy or radiotherapy). If active treatment were to have a large impact on survival, then a patient receiving supportive care would have a shorter survival duration than if he had received active treatment. The initial low PS score given by the oncologist would thus be self-fulfilling. This bias is not obviated by comparing survival in each PS score to that of patients with similar PS scores in other studies, as PS is doctor-assessed in those studies too. However, NSCLC is notoriously poorly responsive to active treatment, so theoretically bias should be minimal when compared to patients with more treatable cancers.

Although patients and oncologists rarely differed by more than one PS point, a few patients gave surprisingly poor scores, even though they appeared to be in good health. The reason behind this variation in scores was not explored in this study, but could be explained by physical or psychological comorbidity that was overlooked by the oncologists. Hopwood and Stephens (2000) demonstrated that 33% patients with lung cancer had self-reported depressive illness, which can negatively impact on their response to treatment (Stommel *et al*, 2002) and was frequently undiagnosed by their oncologists (Ford *et al*, 1994). If patients are routinely asked to assess their own PS, and this is used as a basis for discussion during clinic, concerns (such as comorbidity) may be raised and addressed. Six patients and oncologists disagreed over the PS score by more than 2 points and are listed in Table 5. The numbers are too small from which to draw any statistical conclusions, but among these patients neither the oncologists nor the patients themselves were generally more optimistic in their PS assessment. All six patients had stage III or IV NSCLC at the time of assessment and died before the study was completed.

In this study, patients had not received a confirmed diagnosis of cancer, and it is difficult to estimate how much this contributed to their PS assessment. Ascribing treatment purely on the basis of patient PS assessment could perhaps introduce its own problems with bias. Those with unrealistically optimistic expectations of a trial outcome may overestimate their own PS scores in order to meet trial entry criteria. Equally, those wishing to avoid entering a certain clinical trial may be unduly pessimistic about their PS. However, oncologists could be just as likely to introduce bias, especially with patients on the borderline between PS 1 and 2, the cut off point for many of the combination chemotherapy trials.

In conclusion, PS is a simple and useful tool, either used alone or in the context of a multivariable prognostic test. This study showed that, for patients with NSCLC, PS correlated with overall survival; whether assessed by the patients themselves or their treating oncologists. We showed that patients are reliable assessors of their own PS, although it is not known how much this would differ if they had previously been made aware of their diagnosis. Involving the patient in PS score allocation may not only highlight their concerns and comorbidity, but may also reduce oncologist interobserver variation and sex bias. It would be interesting to extend this trial to include more SCLC patients as well as involving patients with other cancer types.
